# Density, viscosity, speed of sound, flash point, bulk modulus, and surface tension of mixtures of military jet fuel JP-5 and biodiesels dataset

**DOI:** 10.1016/j.dib.2022.107849

**Published:** 2022-01-21

**Authors:** Dianne Luning Prak, Michael Hamilton, Rhea Banados, Jim Cowart

**Affiliations:** a Department of Chemistry, U.S. Naval Academy, 572M Holloway Road, Annapolis, MD 21402, United States; b Department of Mechanical Engineering, U.S. Naval Academy, 590 Holloway Road, Annapolis, MD 21402, United States

**Keywords:** Density, Viscosity, Speed of sound, Flash point, Bulk modulus, Surface tension

## Abstract

In this work, the density, viscosity, speed of sound, flash point, and surface tension of mixtures of military jet fuel JP-5 and biodiesels were measured at various temperatures. The biodiesels were synthesized from various oils (avocado, canola, castor, coconut, corn grapeseed, linseed, neem, oil, palm, peanut, soybean, and walnuts), bacon grease, and duck fat. The isentropic bulk modulus was calculated from the speed of sound and density. These physical properties are important for modeling the spray of fuel into a diesel engine cylinder.

## Specifications Table

**Table:** 

Subject	Chemistry
Specific subject area	Methyl ester biodiesels were synthesized from several oils and the compositions are summarized in Luning Prak et al. [1]. These biodiesels were mixed with military jet fuel, JP-5. The physical properties of these mixtures are reported at several temperatures.
Type of data	Excel table
How the data were acquired	The densities and speed of sounds of the components and their mixtures were measured using an Anton Paar DSA 5000 density and sound analyzer at 15, 20, and 25 °C that was operated under the maximum precision setting. The DSA 5000 uses sound waves at 3 MHz. This instrument was tested daily with degassed ultrapure water, and its density measurements were checked periodically using a NIST-certified toluene density standard (Certificate Standard Reference Material 211d, Toluene liquid density-extended range). The instrument was cleaned between organic liquid samples with hexane and after water samples with ethanol and then dried. The viscosity was measured using an Anton Paar SVM 3000 Stabinger viscometer at 20 and 40 °C that was operated under the maximum precision setting. The SVM 3000 was checked daily with a Paragon Scientific Certified Viscosity reference Standard, APS3. It was cleaned between samples with hexane and dried. The surface tensions were measured using a Kruss DS100 drop shape analyzer was used to measure the surface tension of each individual component or mixture. In drop shape analysis, the computer software fits the Young-LaPlace equation to the shape of a droplet formed on a need tip. The program input includes air density, organic liquid density, and the needle diameter. The diameter was measured with a Mitutoyo micrometer. For each liquid tested, at least three drops were formed, and at least 20 surface tension measurements were taken for each drop. A Setaflash Series 8 closed cup flash point tester model 82,000–0 (Stanhope-Seta) was used in temperature ramping mode to measure flash point. The 82,000–0 model conforms to ASTM D3828 (gas ignition option), ASTM D1655 (gas ignition option), ASTM D3278, ASTM D7236, and ASTM E502, as given in the manufacturer's literature. For all instruments, the measurement for multiple samples of each liquid were taken from which the average and standard deviation were determined.
Data format	Raw Analyzed
Description of data collection	Mixtures of JP-5 and biodiesel were prepared by weighing on an analytical balance at room temperature, 21 °C, in a temperature-controlled (heating and air conditioned) building. The surface tension measurements were taken at room temperature. The viscometer and densitometer controlled the temperature. Two or more samples were analyzed for each instrument. The isentropic bulk modulus was calculated from the density and speed of sound.
Data source location	All samples were prepared and analyzed at • U. S. Naval Academy • Annapolis, MD • USA
Data accessibility	With this article
Related research article	D.J. Luning Prak, M. Hamilton, R. Banadaos, J. Cowart, Combustion and physical properties of blends of military jet fuel JP-5 with fifteen different methyl ester biodiesels synthesized from edible and nonedible oils, Fuel vol. (2022) 10.1016/j.fuel.2021.122503

## Value of the Data

• Researchers who are modeling the combustion behaviors of fuels require density, viscosity, and surface tension values. Spray patterns into the combustion chamber, which have been shown to depend on these variables, impact the timing of combustion.
• Researchers have used bulk modulus to explain ignition delay in engines fueled with biodiesel and diesel. The current data allow can be used to predict ignition delay in engines fueled with biodiesel and JP-5 mixtures.
• Military personnel who are trying to assess if a biodiesel can be mixed with JP-5 and used in a diesel engine can use the data to predict density, viscosity, and flash point.
• Researchers who are developing correlations between biodiesel composition and physical properties can use the compositional information in Luning Prak et al. [1] and the physical properties here. These correlations can help predict the properties of biodiesels synthesized from oil sources.

## 1 Data Description

The data presented in the associated excel file are a more complete data set for a recent article by Luning Prak et al. [1]. This excel file summarizes the measured density (15, 20, 25 °C), viscosity (20 and 40 °C), flash point, and surface tension (21 °C) of biodiesels, JP-5, and their blends. Table 1 compares the measured density and viscosity of the biodiesels with those in the literature. The isentropic bulk modulus, *E_v_,* was calculated at each temperature and ambient pressure by:

(1) $$E_{v}/\text{Pa}=\left(c^{2}\times \rho \right)$$

using the density (*ρ*) and speed of sound (*c*) measurements.

**Table 1:** Comparison of the density and viscosity of the biodiesels measured herein and those reported in the literature.

Fuel	Density (kg·m^−3^) 288.15 K	Literature	Viscosity (mm^2^·s^−1^) 313.15 K	Literature
avocado	880.0	877.68, 875–912 [2,3]	3.93	3.75–10.65, 4.42, 4.9581 [2,^3^,6]
bacon grease	875.3	873.2 [4] (pork lard)	3.81	4.63 [4] (pork lard)
canola	883.2	878, 880–900 [5,7]	4.54	3.53, 4.255, 4.42 [5,^7^,8]
castor	929.3	900, 922, 924, 946.1 [5,[9], [10], [11]	15.8	12.77, 14.08, 15.4 [9], [10], [11]
coconut	879.9	867, 870 [5,12]	3.05	2.726, 3.20 [5,8]
corn	884.8 *881.2 at 293 K	880–890 [7] *881.8 at 293 K [13]	3.74 *6.05 at 293 K	3.39, 4.4 [7,14] *6.3288 at 293 K [13]
duck fat	879.0		3.84	4.363 [8]
grapeseed	885.7	880.2 [15]	4.20	4.04, 4.1 [14,15]
linseed	893.7 *890.0 at 293 K	852 [16] *883.3 at 293 K [13]	3.79 5.14 at 293 K	3.95 [16] *5.8481 and 8.2 at 293 K [13,17]
Neem	885.4	866, 876.2, ∼876, 820–940 [5,^10^,^18^,19]	5.18	4.23, 5.53, 6.09, 3.2–10.7 [5,^10^,^18^,19]
olive	878.7	882.3–887.4 [20]	4.01	4.512, 4.52, 5.29–6.46 [6,^8^,20]
palm	875.4	860–900, 870, 877 [5,^7^,21]	4.58	4.42, 4.43, 4.53, 4.54, 4.698 [5,^7^,^7^,^21^,22]
peanut	879.9	878, 883, 886.4 [5,^7^,22]	4.72	4.42, 4.69, 4.98 [5,^7^,23]
soybean	886.1	882, 885 [5,7]	4.26	2.019, 4.08, 4.08, 4.15, 4.2 [5,^7^,^8^,14]
walnut	888.3		3.47	3.88 [23]

## 2 Experimental Design, Materials and Methods

The synthesis methods for the biodiesels and their compositions are given Luning Prak et al. [1]. Mixtures of JP-5 and biodiesel were prepared by weighing each component on an analytical balance at room temperature, 21 °C. The densities and speed of sounds of the components and their mixtures were measured using an Anton Paar DSA 5000 density and sound analyzer at 15, 20, and 25 °C that was operated under the maximum precision setting. The DSA 5000 uses sound waves at 3 MHz. This instrument was tested daily with degassed ultrapure water, and its density measurements were checked periodically using a NIST-certified toluene density standard (Certificate Standard Reference Material 211d, Toluene liquid density-extended range). The instrument was cleaned between organic liquid samples with hexane and after water samples with ethanol and then dried.

The viscosity was measured using an Anton Paar SVM 3000 Stabinger viscometer at 20 and 40 °C that was operated under the maximum precision setting. The SVM 3000 was checked daily with a Paragon Scientific Certified Viscosity reference Standard, APS3. It was cleaned between samples with hexane and dried.

The surface tensions were measured using a Kruss DS100 drop shape analyzer was used to measure the surface tension of each individual component or mixture. In drop shape analysis, the computer software fits the Young-LaPlace equation to the shape of a droplet formed on a need tip. The program input includes air density, organic liquid density, and the needle diameter. The diameter was measured with a Mitutoyo micrometer. For each liquid tested, at least three drops were formed, and at least 20 surface tension measurements were taken for each drop.

A Setaflash Series 8 closed cup flash point tester model 82,000–0 (Stanhope-Seta) was used in temperature ramping mode to measure flash point of the JP-5, coconut biodiesel, and the mixtures of JP-5 with coconut biodiesel. The flash-no flash setting was used to test all other the biodiesels. The maximum temperature of the instrument is 130 °C. All biodiesels except for the coconut biodiesel did not flash at 130 °C, indicating that their flashpoints are greater than 130 °C. The 82,000–0 model conforms to ASTM D3828 (gas ignition option), ASTM D1655 (gas ignition option), ASTM D3278, ASTM D7236, and ASTM E502, as given in the manufacturer's literature.

For all instruments, the measurement for multiple samples of each liquid were taken from which the average and standard deviation were determined. The expanded uncertainties were calculated by multiplying the standard deviation by 2, which approximates the 95% confidence interval for random errors that are normally distributed. The average pressure for these measurements was 0.102 MPa. The standard uncertainty u is u(temperature) = 0.01 K for all density, viscosity, and speed of sound data, and u(temperature) = 1 K for surface tension data. The expanded uncertainties U are U(pressure) =1 kPa, U(density) = 0.1 kg/m^3^, U(viscosity) = 0.01 mPa•s, U(speed of sound) = 0.7 m/s, U(flash point)= 1 K, and U(surface tension) = 0.2 mN/m, and combined expanded uncertainties *U* are U(bulk modulus) = 1 MPa, *U*(*x_1_*) = 0.0001 for mole fractions less than 0.7 and *U*(*x_1_*) = 0.0002 for the higher mole fractions.

## CRediT authorship contribution statement

**Dianne Luning Prak:** Conceptualization, Formal analysis, Methodology, Investigation, Writing – original draft. **Michael Hamilton:** Investigation. **Rhea Banados:** Investigation. **Jim Cowart:** Investigation, Methodology, Writing – review & editing.

## Declaration of Competing Interest

The authors declare that they have no know competing financial interests or personal relationships that could have appeared to influence the work reported in this paper.

## Appendix Supplementary materials

Image, application 1
